# CUL5-SOCS6 complex regulates mTORC2 function by targeting Sin1 for degradation

**DOI:** 10.1038/s41421-019-0118-6

**Published:** 2019-10-29

**Authors:** Binghai Cui, Liyan Gong, Min Chen, Yuxue Zhang, Huairui Yuan, Jun Qin, Daming Gao

**Affiliations:** 1 State Key Laboratory of Cell Biology, CAS Key Laboratory of Systems Biology, CAS Center for Excellence in Molecular Cell Science, Innovation Center for Cell Signaling Network, Shanghai Institute of Biochemistry and Cell Biology, University of Chinese Academy of Sciences, Chinese Academy of Sciences, 320 Yueyang Road, 200031 Shanghai, China; 2 The Key Laboratory of Stem Cell Biology, CAS Center for Excellence in Molecular Cell Science, Institute of Health Sciences, Shanghai Institutes for Biological Sciences, Chinese Academy of Sciences/Shanghai Jiao Tong University School of Medicine, University of Chinese Academy of Sciences, 200031 Shanghai, China

**Keywords:** Cell signalling, Cancer therapy

Dear Editor,

The mammalian target of rapamycin (mTOR) is a serine/threonine kinase and a central regulator of cell homeostasis by forming two structurally similar but distinct complexes, namely mTORC1 and mTORC2, to phosphorylate different groups of substrates^1^. Composed by Sin1, Rictor, GβL, and mTOR, mTORC2 mainly regulates cell survival and proliferation by phosphorylating hydrophobic motif of AKT (Ser473 site for AKT1) and possible other members of the AGC kinase family^1–3^. As a central modulator of cell signaling network, mTORC2 activity is subjected to various regulations. For instance, T86 and T398 phosphorylation of Sin1 impairs the integrity of mTORC2 complex and suppresses AKT activation, and PtdIns (3,4,5) P3 interacts with PH domain of Sin1 and results in activation of mTORC2 complex^4,5^. Therefore, Sin1 serves as the key component to mediate the cross talk between physiological signals and proper mTORC2 function. Although mTOR and Rictor have been shown to be regulated by ubiquitin E3 ligases, protein degradation-dependent regulation of Sin1 or mTORC2 activity has not been investigated.

Ubiquitin-proteasome system (UPS) is the major system to govern protein turnover by conjugating ubiquitin molecules to protein substrates as signals for proteasome degradation. Cullin-RING ubiquitin-protein ligases (CRLs) are the largest ubiquitin E3 ligase family, and a typical CRL includes a Cullin protein (CUL) serving as scaffold, a substrate recognition subunit, and a Ring protein (RBX1 or RBX2, with intrinsic E3 activity)^6^. So far the best studied Cullin family member is Cullin1, which forms CRL1 (also known as SCF complex) to control the turnover of many key onco-proteins or tumor suppressors including c-Myc, β-Catenin, and p27^7^. On the other hand, Cullin5 (CUL5) mainly associates with suppressor of cytokine signaling (SOCS) box proteins, RING finger protein RBX2, and adaptor proteins complex-ElonginB/C, to form functional CRL5 E3 ligases. Although more than 40 SOCS box proteins have been identified, relatively few substrates have been identified and the functional role CRL5 is largely unknown^8^.

We initially found that both proteasome inhibitor MG132 and NEDD8-activating enzyme inhibitor MLN4924 treatment increased Sin1 protein levels in 293T cells, indicating that Sin1 protein is possibly governed by CRL family E3 ligases (Supplementary Fig. 1a). Interestingly, depletion of RBX2, but not RBX1, significantly increased Sin1 expression (Fig. 1a). Since RBX2 uniquely associates with CUL5, while the other Cullin family members interact to RBX1^8^, we hypothesized that CRL5 complex may be the major E3 ligase modulating Sin1 degradation. Indeed, Sin1 specifically interacted with CUL5 but not CUL2 in the transfection/Co-immunoprecipitation (IP) experiment (Supplementary Fig. 1b). Importantly, depletion of CUL5, RBX2, and ElonginB/C (the adaptor proteins within CRL5 complex), but not other Cullin proteins, all resulted in upregulation of Sin1 (Fig. 1b and Supplementary Fig. 1c). In order to identify the SOCS box protein that specifically mediates CRL5-dependent Sin1 regulation, we performed shRNA based screening (data not shown) and Co-IP experiments, and an interaction was observed between Sin1 and SOCS6, but no other SOCS box proteins were examined (Supplementary Fig. 1d). And the Sin1-SOCS6 interaction was further validated by Co-IP experiment at endogenous level (Fig. 1c). In support of a role of SOCS6 in regulating Sin1 stability, ectopically expressed SOCS6 promoted Sin1 ubiquitination and shortened Sin1 half-life in the Cycloheximide chase experiment, while depletion of endogenous SOCS6 decreased Sin1 ubiquitination (Fig. 1d and Supplementary Fig. 1e–g). By generating various Sin1 truncations, we found that the N-terminal region (1–136 amino acids) of Sin1 is responsible for SOCS6 interaction and SOCS6 dependent poly-ubiquitination (Supplementary Fig. 2a–c). Based on possible Sin1 ubiquitination sites reported by PhosphoSitePlus (https://www.phosphosite.org/), we further constructed various Sin1 mutants, and found that 4 lysine sites (K162, K166, K276, K302) are possibly the major ubiquitination sites of Sin1, since mutation of them reduced Sin1 ubiquitination (Supplementary Fig. 2d). Moreover, among these 4 identified major ubiquitination sites, K162, K276, and K302 possibly mediate SOCS6-dependent ubiquitination and degradation of Sin1, as their mutation effectively blocked ectopic SOCS6-mediated Sin1 downregulation (Supplementary Fig. 2e).

**Fig. 1:**
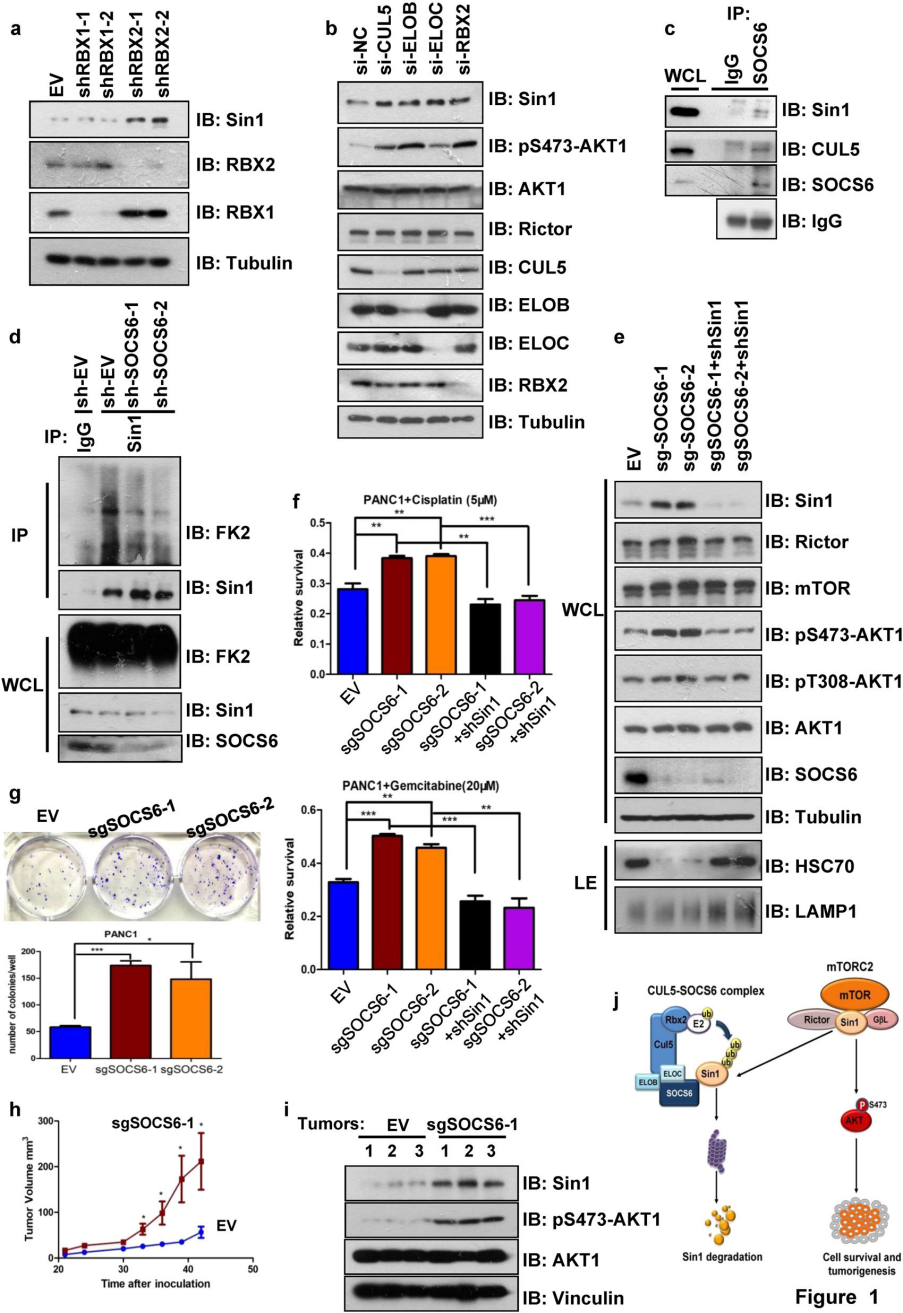
The CUL5-SOCS6 E3 complex promotes Sin1 degradation, and regulates cell survival and tumorigenesis. **a**, **b** Depletion of RBX2 (**a**), ElonginB/C, and CUL5 (**b**) upregulated Sin1 in 293T cells. **c** The interaction of SOCS6 and Sin1 was detected at endogenous level in 293T cells. MG132 was added 12 h prior to cell harvest for immuno-precipitation.WB was detected with indicated antibodies as shown. **d** Knockdown of SOCS6 decreased endogenous Sin1 ubiquitination in 293T cells. Cells were treated with shRNAs targeting SOCS6 and cell lysates were made after MG132 treatment (12 h) for immunoprecipitation and detected with indicated antibodies. FK2 detected ubiquitinated proteins. **e** Lentiviral sgRNAs-mediated SOCS6 knockout (ko) elevated Sin1 expression and subsequently activated mTORC2 in PANC1 cells, while Sin1 knockdown reversed such effect. **f** SOCS6 ko PANC1 cells from (**e**) were more resistant to Cisplatin or Gemcitabine treatment. Data are mean ± SEM (*n* = 3), **P < 0.01, ***P < 0.001, Student’s *t*-test. **g** SOCS6 ko PANC1 cells from (**e**) formed more colonies than control cells in anchorage dependent growth condition. Data are mean ± SEM (*n* = 3), *P < 0.05 ***P < 0.001, Student’s *t*-test. **h**, **i** SOCS6 ko PANC1 cells were more potent in xenograft tumorigenesis assay (**h**), which may be caused by elevated mTORC2-AKT1 pathway (**i**). For (**h**), data are mean ± SEM (*n* = 6), *P < 0.05, Student’s *t*-test. Lysates of 3 tumors from each group were prepared and analyzed in (**i**). **j** The schematic model of how the CUL5-SOCS6 complex regulates mTORC2 function via Sin1 degradation

Next, we continued to investigate whether SOCS6 modulates mTORC2 activity via governing Sin1 expression. Although SOCS6 could not compete with mTOR for Sin1 interaction (Supplementary Fig. 2f), ectopic expression of SOCS6 in HEK293T cells decreased the expression of endogenous Sin1 and pS473-AKT1 signal (representing mTORC2 activity), which could be reversed by addition of proteasome inhibitor MLN2238 (Supplementary Fig. 3a). Since *SOCS6* gene was reported downregulated in pancreatic cancer^9^, we utilized pancreatic cancer cell line PANC1 and BXPC3 to examine the potential impact of SOCS6 to mTORC2-AKT pathway. Ectopic expression of SOCS6 in PANC1 cells reduced the endogenous pS473-AKT1 signal without affecting the AKT1 protein abundance, and decreased cell survival rate after Cisplatin treatment (Supplementary Fig. 3b, c). Knockout of *SOCS6* gene in both PANC1 and BXPC3 cells with CRISPR/Cas9 method resulted in accumulated Sin1 protein, increased pS473-AKT1 signal, and reduced chaperon-mediated autophagy^10^(evidenced by alteration of lysosomal HSC70 levels), but only had minimal effect on mTOR and Rictor, other two essential components of mTORC2 complex (Fig. 1e and Supplementary Fig. 3d). Importantly, the activation of AKT1 in sgSOCS6 cells was effectively reversed by shRNA-mediated Sin1 depletion (Fig. 1e and Supplementary Fig. 3d), further supporting that SOCS6 is a master regulator of mTORC2-AKT pathway. Notably, different from the pS473-AKT1 signal, the PI3K-PDK1 pathway mediated AKT1 Thr308 phosphorylation was merely affected by SOCS6 knockout or Sin1 knockdown, implicating a highly specific regulation of SOCS6 on mTORC2 activity (Fig. 1e and Supplementary Fig. 3d).

The activation of AKT is important for cells to resist to apoptotic signals via various mechanisms, and therefore elevated AKT signaling has been related to drug resistance in cancer cells. To examine whether SOCS6-dependent Sin1 regulation plays a role in cell survival and drug resistance, we treated the resulted PANC1 and BXPC3 cells described above with Cisplatin and Gemcitabine, and determined the cell survival by CCK8 experiments. As indicated, depletion of SOCS6 significantly increased cell survival post drug treatment, which was reversed by knockdown of Sin1 or AKT inhibitor treatment (Fig. 1f and Supplementary Fig. 3e, f). Collectively, these data suggested a crucial function of SOCS6 in regulating cell survival possibly via the mTORC2-AKT axis.

AKT has been implicated in promoting cell proliferation through regulating different substrates including Skp2, which is the master regulator of cell cycle via degrading p21, P27, and many others^7^. Therefore we went on to investigate whether depletion of SOCS6 has any effect on cell proliferative features. Indeed, the sgSOCS6-treated PANC1 cells proliferated much faster than control cells and formed much more colonies in anchorage-dependent conditions (Fig. 1g and Supplementary Fig. 3g). To evaluate the functional impact of SOCS6 in regulating tumorigenesis, SOCS6-depleted or vector-treated PANC1 cells were subcutaneously implanted into nude mice to perform xenograft tumorigenesis assay. Tumors grew in both groups 20 days post inoculation, and the tumor sizes were measured every 3 days. Strikingly, the mice transplanted with SOCS6-depleted PANC1 cells developed much bigger tumors than the mice injected with control PANC1 cells (Fig. 1h and Supplementary Fig. 3h, i). Moreover, the tumors derived from sgSOCS6-treated PANC1 cells had higher pS473-AKT1 signal than the control group, further supporting SOCS6’s role in inhibiting mTORC2-AKT pathway (Fig. 1i, j). In consistent with the putative tumor suppressor function of SOCS6, we found two naturally occurred SOCS6 mutations (K17N, reported in prostate cancer; E61Q, reported in head and neck cancer) reported in COSMIC somatic database (https://cancer.sanger.ac.uk/cosmic/) within its N-terminal Sin1-interacting region, which reduced SOCS6-Sin1 interaction and impaired SOCS6-mediated Sin1 degradation (Supplementary Fig. 4a–d). In summary, our results have identified new regulation of mTORC2 pathway by CUL5-SOCS6 E3 complex via Sin1 stability control, and it may shed new light on the understanding of the dynamic signaling cascades involved in cancer cell survival and drug resistance.

## Supplementary information

Supplementary information

## Supplementary information

**Supplementary Information** accompanies the paper at (10.1038/s41421-019-0118-6).

## References

[1] Mossmann D, Park S, Hall MN (2018). mTOR signalling and cellular metabolism are mutual determinants in cancer. Nat. Rev. Cancer.

[2] Ikenoue T, Inoki K, Yang Q, Zhou X, Guan KL (2008). Essential function of TORC2 in PKC and Akt turn motif phosphorylation, maturation and signalling. EMBO J..

[3] Jacinto E (2006). SIN1/MIP1 maintains rictor-mTOR complex integrity and regulates Akt phosphorylation and substrate specificity. Cell.

[4] Liu P (2013). Sin1 phosphorylation impairs mTORC2 complex integrity and inhibits downstream Akt signalling to suppress tumorigenesis. Nat. Cell Biol..

[5] Liu P (2015). PtdIns(3,4,5)P3-dependent activation of the mTORC2 kinase complex. Cancer Discov..

[6] Petroski MD, Deshaies RJ (2005). Function and regulation of cullin-RING ubiquitin ligases. Nat. Rev. Mol. Cell Biol..

[7] Wang Z, Liu P, Inuzuka H, Wei W (2014). Roles of F-box proteins in cancer. Nat. Rev. Cancer.

[8] Okumura F, Joo-Okumura A, Nakatsukasa K, Kamura T (2016). The role of cullin 5-containing ubiquitin ligases. Cell Div..

[9] Arias E (2015). Lysosomal mTORC2/PHLPP1/Akt regulate chaperone-mediated autophagy. Mol. Cell.

[10] Wu K (2013). MicroRNA-424-5p suppresses the expression of SOCS6 in pancreatic cancer. Pathol. Oncol. Res..

