# Supplementary material for: CUL5-SOCS6 complex regulates mTORC2 function by targeting Sin1 for degradation
Source: Cell Discov. 2019 Oct 29;5:52. doi: 10.1038/s41421-019-0118-6 (PMC6868212; doi:10.1038/s41421-019-0118-6)
Supplement: Supplementary file 1 — Supplementary information [file 41421_2019_118_MOESM1_ESM.pdf]

Supplementary information

Materials and methods

Antibodies and plasmids

Primary antibodies used in this study are as follows: anti-mTOR (#2283, Cell Signaling Technology), anti-Rictor (#9476, Cell Signaling Technology), anti-FK2 (#04-263, MILLIPORE), anti-pS473-AKT (#4051, Cell Signaling Technology), anti-pS308-AKT(#4056, Cell Signaling Technology), anti-AKT(#9272,CellSignalingTechnology), anti-Sin1(#05-1044, Millipore),anti-SOCS6(A9957, ABclonal), mouse anti-FLAG tag (F3165, Sigma), rabbit anti-FLAG tag (F7425, Sigma), anti-Tubulin (SC23948, Santa Cruz Biotechnology), mouse anti-HA tag (SC7392,Santa Cruz Biotechnology), rabbit anti-HA tag (51064-2-AP, Proteintech), anti-His tag(AE003,abclonal), anti-Myc tag(#2278, Cell Signaling Technology). The HA-Sin1 was cloned into pcDNA3.1 vector, the FLAG-Sin1 and different deletion mutants of FLAG-Sin1 were cloned into pCMV-FLAG vectors. The HA-SOCS6, FLAG-SOCS6 and different deletion mutants of FLAG-SOCS6 were cloned into pLEX-MCS. shRNAs against target genes were cloned into pLKO.1 vector. The pX330 vector was used to construct sgRNA to knock out SOCS6.

Cell culture and transfection

PANC1, HEK293T, and HeLa cells were cultured in DMEM medium, Bxpc3 cells were cultured in RPMI 1640 medium. All media were supplemented with 10% FBS, 100 units of penicillin/streptomycin. Plasmids were transiently transfected with polyethylenimine (Sigma).

Western blotting and Immunoprecipitation (IP)

Cells were lysed in EBC lysis buffer (50mM Tris-HCl, pH 8.0, 120 mM NaCl, 0.5% Nonidet P-40) with protease inhibitors (Selleck Chemicals) and phosphatase inhibitors (Selleck Chemicals). Protein samples were quantified by using Bio-Rad protein assay kit in a spectrophotometer (Thermo Scientific), separated by SDS-PAGE gel and electro transferred onto a PVDF membrane. After blocking with 5% nonfat dried milk, the membranes were blotted with primary antibodies. For immunoprecipitation, cell lysates were incubated with anti-FLAG M2 agarose beads or anti-HA agarose beads for 3h. For endogenous SOCS6 immunoprecipitation, the cell lysate was pre-cleared with protein A/G plus Sepharose.2μg of SOCS6 antibody was incubated with pre-cleared cell lysates overnight followed by incubation with protein A/G plus Sepharose for 1 h.

CCK8 cell survival assay

For cell survival assay, 3,000 cells/well were plated in 96-well plates. Cells were cultivated with mediums supplemented with 2% FBS. After 24h, cells were treated with different concentrations of cisplatin (Selleck, S1166) or gemcitabine (Selleck, S1714) for 24h. Then CCK8 (meilunbio, MA0218) was added to the plates, 4h later in 37℃, OD was read in a BioTek Eon Multi-Mode Microplate Readers.

Colony formation

PANC1 cells were cultivated with DMEM medium supplemented with 2% FBS. The cells were treated with 1μM cisplatin for 24h. After one week, colonies were stained with crystal violet and counted.

RNA interference, lentiviral shRNA and sgRNA

Cells were transfected with siRNA using X-tremeGENE siRNA Transfection Reagent (Roche) at 50 nM final concentration according to the manufacture's protocol. All the siRNAs were synthesized by Biotend Company, and two independent siRNA duplexes were designed and mixed for transfection to deplete each target gene. All the shRNAs were constructed with pLKO.1 vector. sgRNAs targeting human SOCS6 were constructed by inserting oligonucleotides into the pX330 vector. The siRNAs sequences targeting CUL1 are as follows: (1) GGAUGAGAGUGUACUGAAA; (2) CCAGAUAUAUGGAGCUCUA. The siRNAs sequences targeting CUL2 are as follows: (1) GGCAAAUAUGUACGUCUUA; (2) GAAGAACAAGUACUUGUUA. The siRNAs sequences targeting CUL3 are as follows: (1) AAGGUGCGAGAAGAUGUATT; (2) AACAACUUUCUUCAAACGCUA. The siRNAs sequences targeting CUL4A are as follows: (1) GGAAGAGACUAAUUGCUUA; (2) GAACAGCGAUCGUAAUCAA. The siRNAs sequences targeting CUL5 are as follows: (1) GGCUAAUAGAGCACAAAUA; (2) CCAGCUGAUUCAGUUAAUA. The siRNAs sequences targeting ELOB are as follows: (1) UGAACAAGCCGUGCAGUGA; (2) AGCGGCUGUACAAGGAUGA. The siRNAs sequences targeting ELOC are as follows: (1) AAACCAAUGAGGUCAAUUU; (2) CGUACAAGGUUCGCUACACUA. The siRNAs sequences targeting RBX1 are as follows: (1) GGGAUAUUGUGGUUGAUAA; (2) AGGUG UGUCCAUUGGACAA. The siRNAs sequences targeting RBX2 are as follows: (1) ACAAGAUGUU CUCCCUCAA; (2) CGUGGAAGACGGAGAGGAA. sgRNAs sequences targeting SOCS6 are as follows: (1) CGGAGCGACGTGGACCTTAT; (2) A CGAGCGCACCTGCATGAAC;(3) GTCCAGCCCTGAATGGCGTC (4) GACGTATAACAAAACGACAC. shRNA sequences targeting SOCS6 are as follows: (1) GCCTGTCGTTATTGGACTTAT; (2) GAGCACTCAAATGGTAGGTTT. shRNAs sequences targeting RBX1 are as follows: (1) CTTTCCCTGCTGTTACCTAAT; (2) ATGTC AAGCTAACCAGGCGTC. shRNAs sequences targeting RBX2 are as follows: (1) CCTGTGGGTGAAACAGAACAA; (2) CGACAAGATGTTCTCCCTCAA. shRNA sequence targeting Sin1 is as follows: TAGGTACAACAGCAACCAAGA.

Ubiquitylation assay

HEK293T cells were transfected with the indicated plasmids. 10μM MG132 were added to the cells for 12h. Cells were lysed in EBC lysis buffer, then added agarose beads with antibody anti-HA. The mixtures were incubated for 4h before washed with NETN buffer (20mM Tris-HCl, pH 8.0, 100 mM NaCl, 0.5% Nonidet P-40, 1mM EDTA). The samples were added SDS–PAGE loading buffer, and examined by immunoblotting.

Lysosome protein extraction

PANC1 cells infected with sgRNA lentivirus targeting SOCS6 and shRNA lentivirus targeting Sin1 were cultured and treated with the lysosome protein extraction kit (BB-314521-50T, BestBio) as directions. The CMA component (hsc70) of lysosomes protein was measured with western blotting.

PANC1 xenograft tumorigenesis assay

PANC1 cells were infected Lenti-Cre-Cas9-sgRNA (control or SOCS6). 5×10^6 PANC1 cells in 100 μl PBS were injected subcutaneously into the flank of 6-week-old male BALB/c nude mice. Tumor growth was monitored for 6 weeks by measuring every 3 days. Tumor volume was calculated by the formula: $L\times W\times W/2$.

Supplementary Figures and Legends

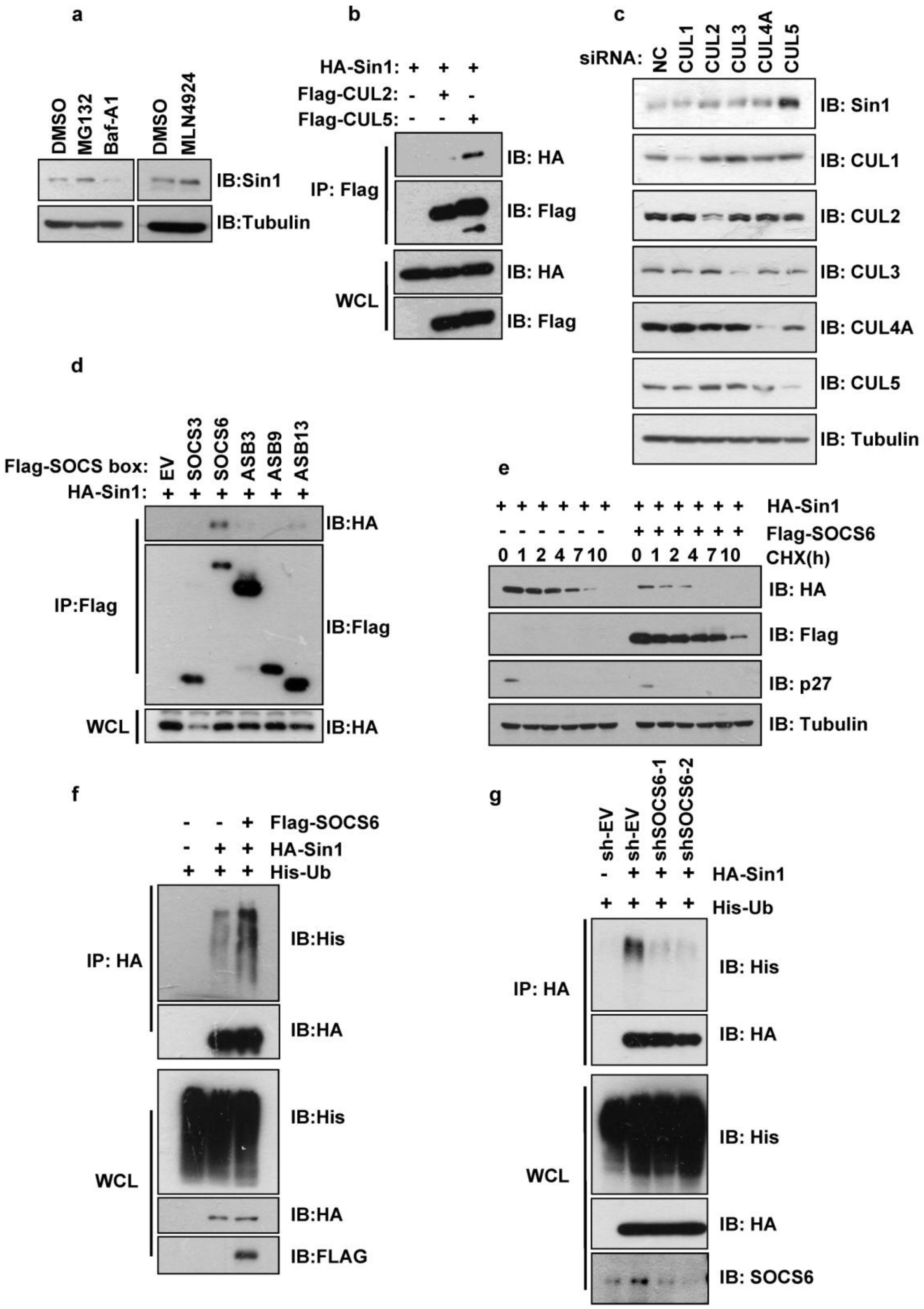

Figure S1. CUL5-SOCS6 E3 complex interacts with and promotes Sin1 ubiquitination and degradation. **(a)** 293T cells were treated with 50 μM MG132 (left panel) or 1 μM MLN4924

(right panel) for 5h, and the cell lysates were made for western blot. **(b)** Immunoblot analysis of Flag-IP and whole cell lysates (WCL) from 293T cells co-transfected with the indicated plasmids. **(c)** 293T cells were transfected with siRNAs targeting various Cullins, and the whole cell lysates were analyzed by western blot with the indicated antibodies. **(d)** 293T cells were co-transfected with the indicated plasmids encoding Sin1 and various SOCS box proteins, and the Flag-IP was carried out and resolved for IB analysis. **(e)** 293T cells transfected with HA-Sin1 in the presence and absence of Flag-SOCS6 were treated with cycloheximide, and collected at the indicated times for western blot. **(f)** 293T cells were transfected with the indicated plasmids, and HA-IP was performed and analyzed by western blot. **(g)** 293T cells were transfected with indicated plasmids and shRNAs targeting SOCS6, and cell lysates were made after MG132 treatment for HA-IP and immune blot.

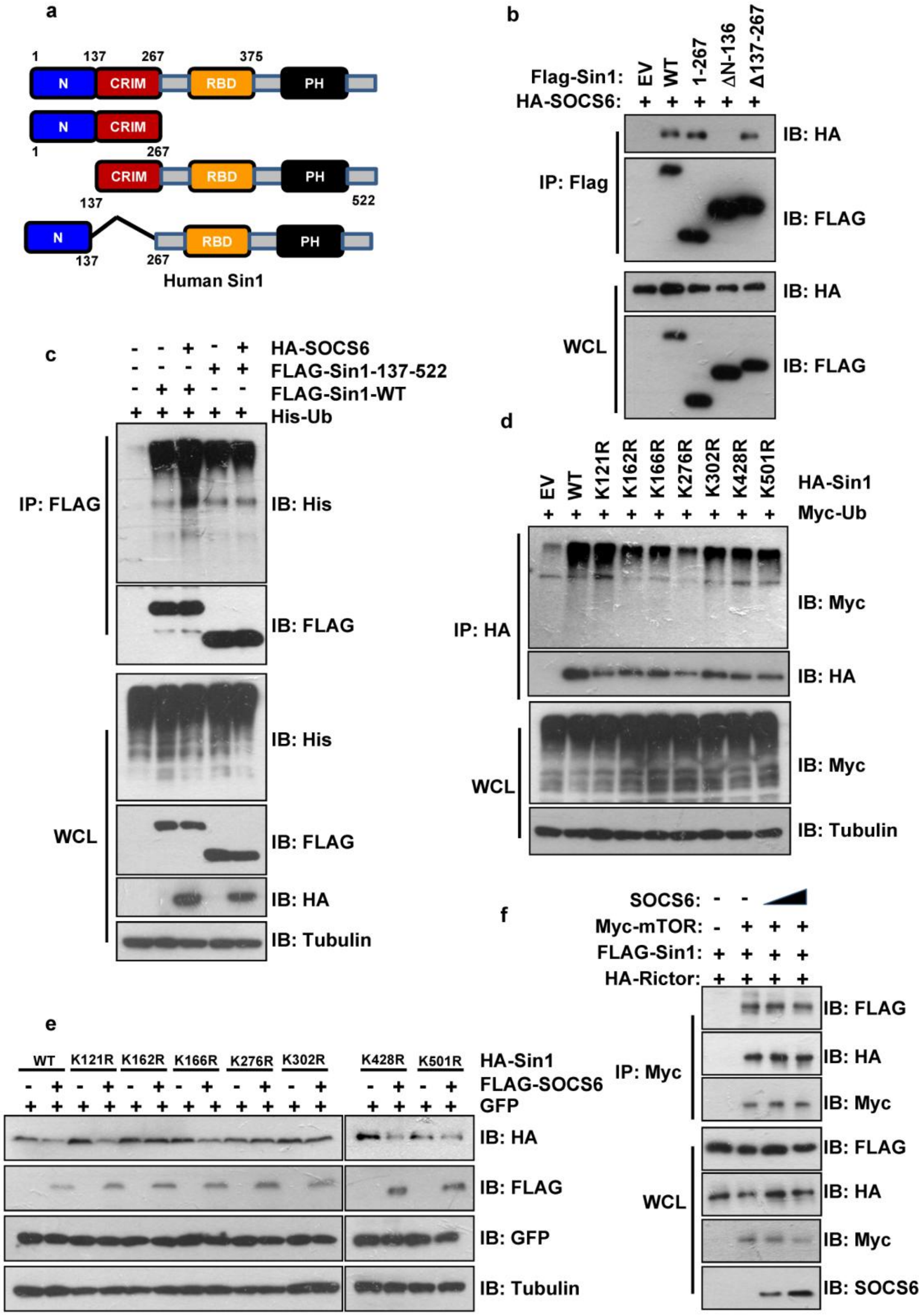

Figure S2. SOCS6 interacts with Sin1 N-terminal region. **(a, b)** Various Sin1 truncates were generated and their interaction with SOCS6 were examined by transfection-IP experiment in 293T cells. MG132 was added 12hrs prior harvest. **(c)** 293T cells were transfected with the

indicated plasmids, and FLAG-IP was performed and analyzed by western blot. MG132 was added 12hrs prior harvest. **(d, e)** Various Sin1 mutants were generated based on possible Sin1 ubiquitination sites reported by PhosphoSitePlus (https://www.phosphosite.org/) and then were co-transfected with Myc-ubiquitin or SOCS6 constructs to examine their ubiquitination status **(d)** and degradation by SOCS6 **(e).** MG132 was added 12hrs prior harvest in **(d)**. **(f)** 293T cells were transfected with the indicated plasmids, and Myc-IP was performed and analyzed by western blot. Increasing amount of SOCS6 expression had no effect on the binding between Sin1 and Rictor-mTOR. MG132 was added 12hrs prior harvest.

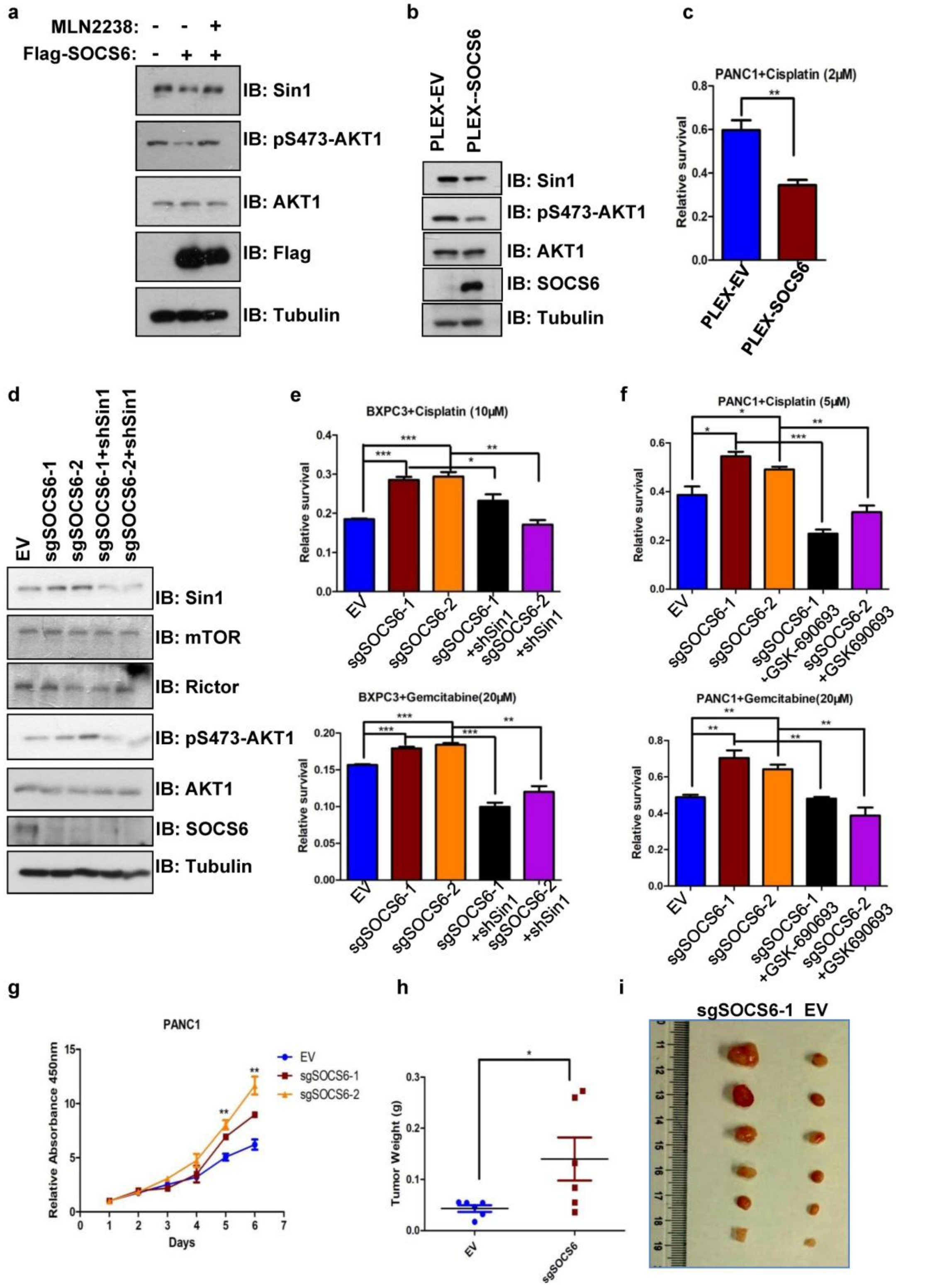

Figure S3. SOCS6 regulates mTORC2-AKT pathway via modulating Sin1. **(a)** 293T cells transfected with Flag-SOCS6 were treated with 10 μM MLN2238 for 5h. The whole cell lysates were analyzed by western blot with the indicated antibodies. **(b)** PANC1 cells infected

with lentivirus encoding SOCS6 were lysed, and the whole cell lysates were analyzed by western blot with the indicated antibodies. **(c)** PANC1 cells from **(b)** were treated with the indicated concentration of cisplatin for 24h, and cell survival was analyzed. Data are mean ± SEM (n = 3), **P < 0.01, Student's t-test. **(d)** BXPC3 cells infected with sgRNA lentivirus targeting SOCS6 and shRNA lentivirus targeting Sin1 were harvested, and the whole cell lysates were analyzed by western blot with the indicated antibodies. **(e)** BXPC3 cells from **(d)** were treated with the indicated concentration of cisplatin or gemcitabine for 24h, and cell survival was analyzed. Data are mean ± SEM (n = 3), *P < 0.05, **P < 0.01, ***P<0.001, Student's t-test. **(f)** SOCS6 ko PANC1 cells from **Fig.1e** were treated with the indicated concentration of cisplatin or gemcitabine with or without the AKT inhibitor-GSK -690693, and cell survival was analyzed. Data are mean ± SEM (n = 3), *P < 0.05, **P < 0.01, ***P<0.001, Student's t-test. **(g)** SOCS6 ko PANC1 cells from **Fig.1e** were cultured in 96 well plate, and the growth curves were measured by CCK8 assay. Data are mean ± SEM (n = 3), **P < 0.01, Student's t-test. **(h, i)** PANC1 cells treated with sgRNA targeting SOCS6 or empty vector were inoculated in the flank of nude mice for xenograft tumorigenesis assay. The tumor weights were measured and analyzed. Data are mean ± SEM (n = 6), *P < 0.05, Student's t-test.

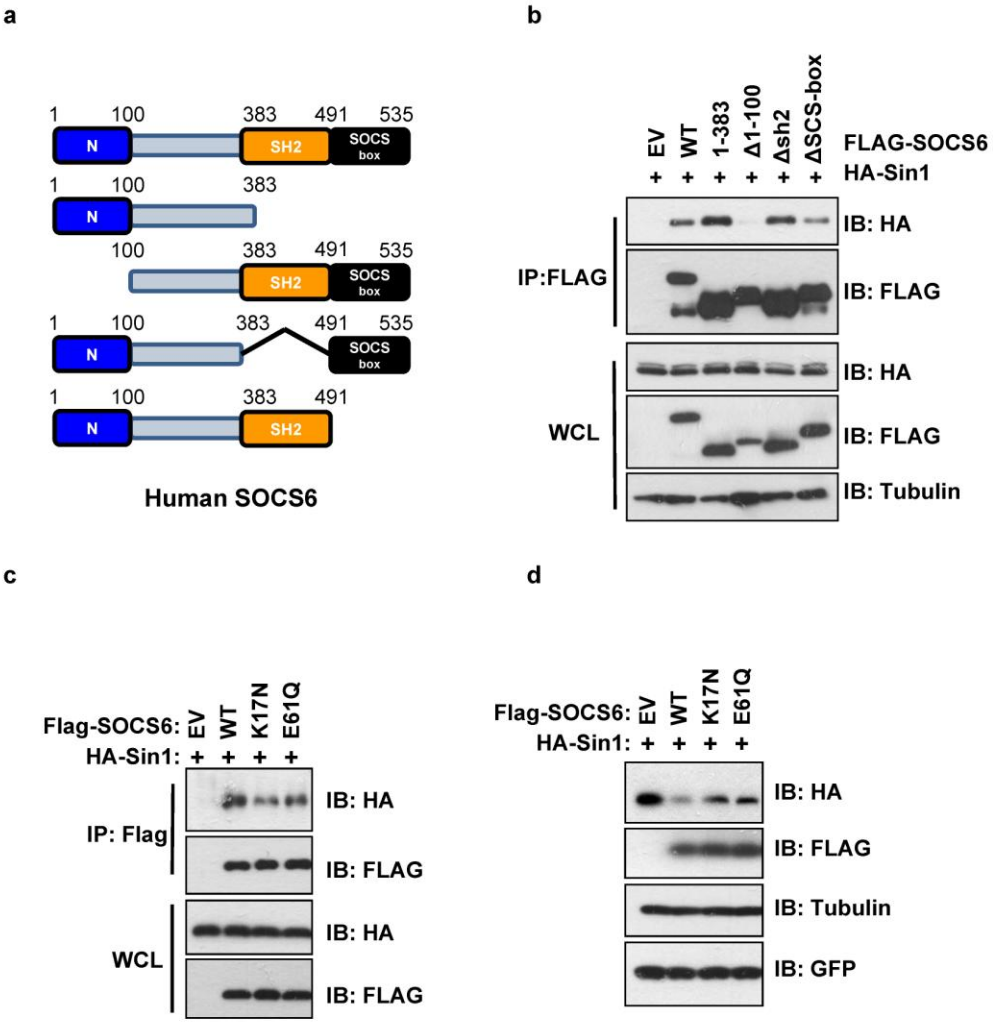

Figure S4. SOCS6 interacts with Sin1 via its N-terminal region. **(a, b)** Various SOCS6 truncates were generated and their interaction with Sin1 were examined by transfection-IP experiment in 293T cells. MG132 was added 12hrs prior harvest. **(c, d)** Based on COSMIC somatic mutation database (https://cancer.sanger.ac.uk/cosmic/), SOCS6-K17N (reported in prostate cancer) and SOCS6-E61Q (reported in head and neck cancer) mutants were generated. The constructs were co-transfected with Sin1 plasmid to examine their capacity to interact with **(c)** and degrade Sin1 **(d)**. MG132 was added 12hrs prior harvest in **(c).** both K17N and E61Q mutations reduced Sin1 interaction and failed to degrade Sin1.
